# Variants in Vitamin D Binding Protein Gene Are Associated With Gestational Diabetes Mellitus

**DOI:** 10.1097/MD.0000000000001693

**Published:** 2015-10-09

**Authors:** Ying Wang, Ou Wang, Wei Li, Liangkun Ma, Fan Ping, Limeng Chen, Min Nie

**Affiliations:** From the Department of Endocrinology, Peking Union Medical College Hospital, Peking Union Medical College, Chinese Academy of Medical Sciences, Beijing, China (YW, OW, WL, FP, MN); Key Laboratory of Endocrinology, Ministry of Health, Beijing, China (YW, OW, WL, FP, MN); People's Hospital of Longkou City, Shan Dong, China (YW); Department of Obstetrics and Gynecology, Peking Union Medical College Hospital, Peking Union Medical College, Chinese Academy of Medical Sciences, Beijing China (LM); and Department of Nephrology, Peking Union Medical College Hospital, Peking Union Medical College, Chinese Academy of Medical Sciences, Beijing, China (LC).

## Abstract

To investigate whether single nucleotide polymorphisms (SNPs) within 4 representative genes (*VDR*, *GC*, *CYP2R1*, and *CYP24A1*) encoding the core proteins involved in vitamin D production, degradation, and ligand-dependent signaling pathway are associated with gestational diabetes mellitus (GDM) in a Chinese population.

A total of 1494 pregnant Han Chinese women (692 women with GDM and 802 women with normal glucose served as controls) were recruited through a 2-step approach. Participants were further divided into 2 groups according to body mass index before gestation (pre-BMI) (25 kg/m^2^). Nine SNPs (rs3733359, rs2282679, and rs16847024 in *GC*, rs2060793 and rs10741657 in *CYP2R1*, rs2248359 and rs6013897 in *CYP24A1*, rs11574143 and rs739837 in *VDR*) were genotyped using TaqMan allelic discrimination assays. The relationships between genotypes/alleles of a single locus as well as haplotypes of each gene and GDM were analyzed.

We did not observe a significant difference in genotype frequency of each SNP between cases and controls. However, in the obese subgroup (pre-BMI ≥ 25 kg/m^2^), the risk allele-A of rs3733359 showed an association with increased risk of GDM (OR = 1.739, 95% CI = 1.066–2.837, *P* = 0.027). The GG-haplotype frequency of rs3733359 and rs2282679 in *GC* was modestly lower in the GDM group (OR = 0.848, 95% CI = 0.719–0.999, *P* = 0.048). Rs2060793 and rs10741657 were associated with insulin area under the curve (*P* = 0.028, *P* = 0.042, respectively), while rs739837 and rs6013897 demonstrated a correlation with fasting glucose (*P* = 0.019, *P* = 0.049, respectively). Additionally, rs2248359 displayed an association with leukocyte counts (B = 0.063 *P* = 0.033) and rs16847024 was related to high-sensitivity C-reactive protein levels (B = 0.086, *P* = 0.005).

Our results indicate an association between *GC* variants and GDM, as well as a relation between a subset of loci in *CYP2R1*, *CYP24A1*, and *VDR* and clinical parameters related to GDM. Our findings may provide information for identifying biomarkers for early risk prediction of GDM and the pathways involved in disease progression.

## Abstract 2

Supplemental Digital Content is available in the text

## INTRODUCTION

The vitamin D endocrine system plays an important role in mineral homeostasis and in regulation of bone remodelling.^1^ In addition to its important roles in physiological processes, the vitamin D endocrine system also participates in pathological processes, including cardiovascular disease, autoimmune disorders, and type 2 diabetes mellitus (T2DM).^2,3^ In humans, only a small amount of vitamin D is obtained through dietary intake, while a vast majority of vitamin D is synthesized in the skin via photochemical conversion of 7-dehydrocholesterol to pre-vitamin D3, and the latter is sequentially metabolized in the liver and kidneys.^4^ The biologically active form of vitamin D, 1,25-dihydroxyvitamin D3 (1,25(OH)_2_D_3_), is mainly produced by 2 hydroxylases: 25-hydroxylase in the liver and 1-alpha-hydroxylase in the kidney. The former is encoded by *CYP2R1* and the latter is encoded by *CYP27B1.*^5^*CYP24A1*, which is transcriptionally induced in vitamin D target cells by the action of 1α, 25-(OH)_2_D_3_, plays an important role in the inactivation pathway from 1α,25-(OH)_2_D_3_ to calcitroic acid.^5^ Additionally, the vitamin D-binding protein (group-specific component protein, GC), which serves to transport vitamin D and deliver circulating vitamin D to target tissues, is specifically responsible for vitamin D endocytosis.^6^ Vitamin D receptor (VDR), a member of the steroid/thyroid hormone receptor family that functions as a transcriptional activator of numerous genes, is essential for vitamin D activity in target tissues.^7^ Recently, multiple loci in *VDR*, *GC*, *CYP2R1*, *CYP24A1*, *CYP27B1*, and *CYP27A1* genes were found to be associated with vitamin D levels.^8–10^

Gestational diabetes mellitus (GDM) is defined as glucose intolerance, with its onset or first recognition during pregnancy.^11^ Numerous studies have suggested that vitamin D deficiency contributes to decreased insulin secretion and the resultant abnormal glucose tolerance in pregnant women,^12–14^ and administration of vitamin D reduces fasting glucose concentration in part by altering insulin sensitivity in women with GDM.^15^ Pregnant women require higher levels of vitamin D in order to meet the calcium requirements of the growing fetus.^16^ However, both diabetic mothers and their fetuses are known to be at greater risk of vitamin D insufficiency compared with nondiabetic pregnant women.^17^

Vitamin D plays important roles in β-cell function and impaired glucose tolerance in GDM, so it is plausible that common variants in the genes that influence vitamin D levels could predispose to GDM. To date, few studies have confirmed such an association,^18,19^ presumably due to lack of statistical power, a small effect size of common variants, or ethnic heterogeneity among different populations. In this study, we selected 9 single nucleotide polymorphisms (SNPs) within 4 representative genes (*VDR*, *GC*, *CYP2R1*, and *CYP24A1*) encoding the core proteins involved in vitamin D production, degradation, and ligand-dependent signaling, in order to evaluate a potential relationship between these genetic variants and GDM.

## SUBJECTS AND METHODS

### Subjects

A total of 1494 unrelated Chinese Han pregnant women were recruited from Peking Union Medical College Hospital in Beijing between 2006 and 2010. All subjects without a previous diagnosis of glucose intolerance were routinely offered a 50 g glucose challenge test (GCT) at 24 to 28 weeks of pregnancy. A plasma glucose concentration of 7.8 mmol/L (1 hr after glucose intake) or more was considered positive for GCT and was followed by a 100 g 3-hr oral glucose tolerance test (OGTT). GDM was defined according to the diagnostic criteria accepted by the American Diabetes Association which has glucose values 2 or higher than the threshold values during the 100 g OGTT (the threshold glucose values were 5.3, 10.0, 8.6, and 7.8 mmol/L at 0, 1, 2, and 3 hr, respectively). The subjects with glucose values all below the threshold were diagnosed as normal glucose tolerance (NGT). Based on the above definition, 692 GDM subjects and 802 NGT control subjects were included.

Written informed consent was obtained from each subject. The study protocol was approved by the Research and Ethics Committee of Peking Union Medical College Hospital.

### Clinical Measurements

The age, height, weight, and blood pressure of all subjects at the 24 to 28 weeks of gestation, and family history of T2DM in the first-degree relatives of subjects were recorded. BMI before gestation (pre-BMI) was calculated as body weight (kg)/square of height (m^2^). In addition, fasting plasma glucose (FPG), fasting plasma insulin (FPI), glycated hemoglobin, high sensitivity C-reactive protein (hs-CRP), white blood cell counts, and platelet counts were measured.

To evaluate basal insulin resistance, we used the insulin resistance index derived from the homeostatic model assessment (HOMA) calculated according to the following equation: HOMA-IR (homeostasis model assessment of insulin resistance) = (FPI in mU/mL × FPG in mmol/L)/22.5. HOMA-B (homeostasis model assessment of β-cell function) and insulin area under curve (AUC) during a 100 g OGTT were applied to assess β-cell function. HOMA-B was calculated according to the formula: (FPI in mU/mL × 20)/(FPG in mmol/L − 3.5). Total AUC of insulin was obtained from the trapezoid method as: V1 + V2 + 0.5 × V0 + 0.5 × V3, where V is the insulin concentration at the indicated time.

Hs-CRP, white blood cell, and platelet count were selected as the parameters of low-grade inflammation, consistent with previous studies.^20^

### SNP Selection, Genotyping, and Genotype Quality Control

We selected 4 genes (*VDR*, *GC*, *CYP2R1*, and *CYP24A1*) related to the production, degradation, and ligand-dependent signaling of vitamin D. Based on the screening standards (the minor allele frequencies are more than 20% in Han Chinese according to the HapMap CHB group, available at http://snp.cshl.org/cgi-perl/gbrowse/hapmap22_B36/), 9 loci were identified, including rs3733359, rs2282679, and rs16847024 in *GC*, rs2060793 and rs10741657 in *CYP2R1*, rs2248359 and rs6013897 in *CYP24A1*, and rs11574143 and rs739837 in *VDR*. Detailed information on the 9 loci is shown in the Table S1, http://links.lww.com/MD/A443.

All polymorphisms were genotyped using Taqman allelic discrimination assays. Allelic discrimination assays were prepared as 5 μL reactions in 384-well plates containing 2.5 μL of 2× Taqman Universal Master Mix (Applied Biosystems, Foster City, California, USA), 0.125 μL of 40× Assay Mix including forward and reverse primers and FAM and VIC labeled probes, 10 to 20 ng of genomic DNA, and distilled H_2_O. The 384-well plates were then placed in a thermal cycler of the VIIA™ 7 instrument (Applied Biosystems), heated for 2 min at 50°C, denatured at 95°C for 5 min and cycled at 95°C for 15 sec and 60°C for 1 min, for a total of 40 cycles. The data output was subsequently processed automatically and analyzed with ViiA™ 7 Software v1.1. Genotyping quality controls were performed in 10% of the samples by duplicate assaying (rate of concordance in duplicates >99%) and the genotyping success rate was similar for cases and controls, with an overall call rate of 96.53%.

### Statistical Analysis

Quantitative variables with normal distributions (platelet count) are presented as mean ± standard deviation (SD) while variables with non-normal distributions are presented as medians and interquartile ranges. The continuous data with normal distributions or log-transformed variables (HOMA-B, HOMA-IR, and AUC of insulin) were analyzed by *t* test. Nonparametric tests were performed to analyze the other variables.

The Hardy–Weinberg equilibrium at individual loci was assessed by χ^2^ tests before association analysis. Odds ratios (OR) with 95% confidence intervals (CI) were determined to describe the strength of association using a logistic regression model, adjusting for pre-BMI, and family history of type 2 diabetes in a first-degree relative as confounding factors.^21^ The determination of the confounding factors is as follows. We firstly did correlation analysis between dependent (eg, fasting glucose) and independent variants (SNP loci and clinical indexes listed in Table S2, http://links.lww.com/MD/A443) and also between SNP loci and clinical indexes to make sure which clinical index might be a confounder.^22^ We found that pre-BMI and first-degree relative correlated with both fasting glucose and SNP loci. Quantitative traits were analyzed by linear regression adjusted for pre-BMI, and the regression coefficients (B) were presented. All *P* values were 2 sided, and differences were considered statistically significant when *P* < 0.05. Statistical analyses were performed using SPSS 11.0 (SPSS, Inc., Chicago, IL). Haplotypes were analyzed using SHEsis software, available at http://analysis.Bio-x.cn/myanalysis.php.

### Power Calculations

Statistical power was analyzed with the Genetic Power Calculator, available at http://ibgwww.colorado.edu/∼pshaun/gpc/. In the power calculation, the prevalence of GDM was assumed to be 3% and the high-risk allele frequency was 0.20. Under a multiplicative model, our present study (a sample of 692 cases and 802 controls) had a power >80% to detect an effect size of 1.3 with a type I error rate of 0.05.

## RESULTS

The clinical characteristics of participants are summarized in Table S2, http://links.lww.com/MD/A443. Compared with controls, the mean age, pre-BMI, systolic blood pressure, diastolic blood pressure, FPG, FPI, glycated hemoglobin, and HOMA-IR were significantly higher in the GDM group (*P* < 0.001), whereas the levels of HOMA-B and AUC of insulin were significantly lower in the GDM group (*P* < 0.001).

### Genotype Analysis

All loci conformed to Hardy–Weinberg equilibrium as shown in Table S1, http://links.lww.com/MD/A443. We did not observe a significant difference in genotype frequency of each SNP between cases and controls (Table S3, http://links.lww.com/MD/A443). To adjust the effect of obesity, we divided the participants into 2 subgroups based on pre-BMI: nonobese (pre-BMI < 25 kg/m^2^) and obese (pre-BMI ≥ 25 kg/m^2^). The risk allele-A of rs3733359 was found to be associated with GDM in the obese group (OR = 1.739, 95% CI = 1.066–2.837, *P* = 0.027), but not in the nonobese group (OR = 0.980, 95% CI = 0.812–1.183, *P* = 0.836) as shown in Table 1. No significant difference was found at other loci.

**TABLE 1:**
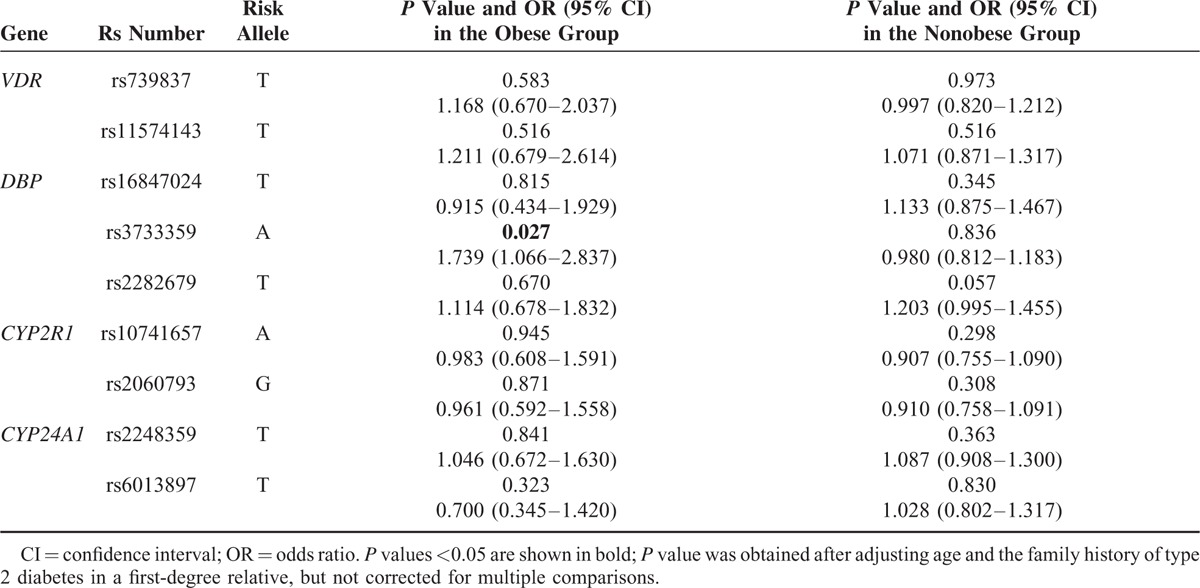
Genotype Distribution and Corresponding Odds Ratios for Gestational Diabetes Mellitus in the Obese and Nonobese Subgroups

### Haplotype Analysis

Linkage disequilibrium (LD) analysis demonstrated that SNPs in *CYP2R1* and *VDR*, but not in *CYP24A1*, existed at the same LD, respectively. Three polymorphisms in *GC* were not completely at the same LD (D′ from 0.58 to 0.83, r^2^ from 0.023 to 0.204). The haplotype frequency distribution of each gene between GDM and controls is summarized in Table 2. The GG-haplotype frequency of rs3733359 and rs2282679 in *GC* was marginally lower in women with GDM (OR = 0.848, 95% CI = 0.719–0.999, *P* = 0.048). The frequencies of 2 additional haplotypes (TA and TG) were similar between the controls and cases (*P* = 0.99 and *P* = 0.065, respectively). No association was observed between GDM and haplotypes in either *VDR* or *CYP2R1.*

**TABLE 2:**
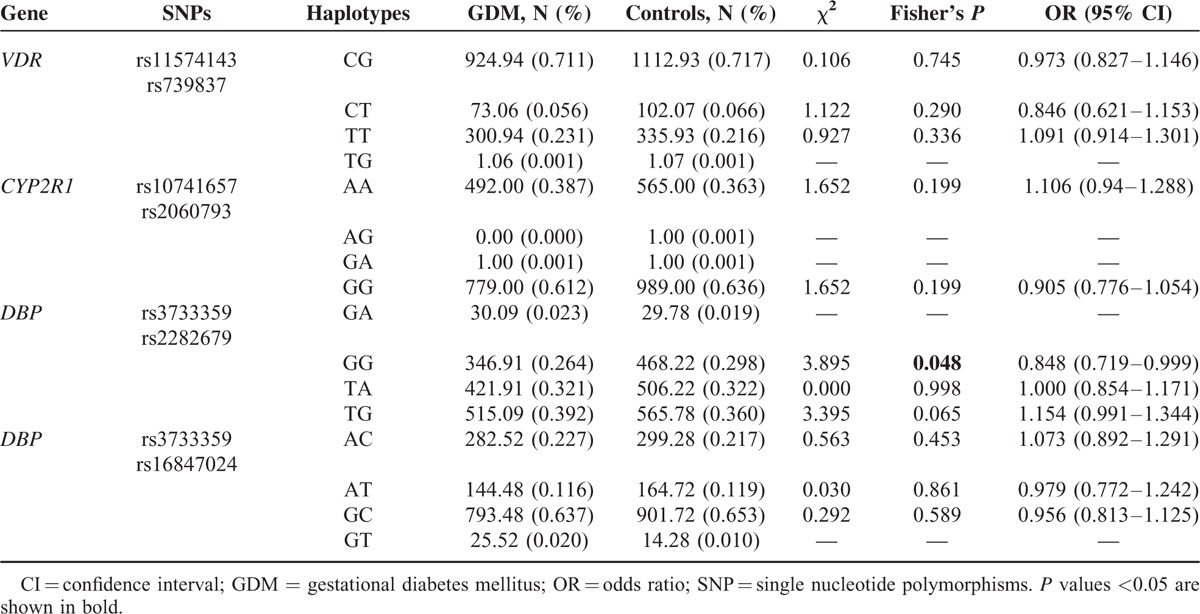
Haplotype Analysis of the Genes Between GDM and Controls

### Genotype–Phenotype Analysis

We performed genotype–phenotype association for the 9 loci, and found that rs739837 in *VDR* showed relation with FPG after adjusting for pre-BMI (*P* = 0.019, Table 3). The per-risk-allele shift in FPG was 0.065 mmol/L. Each individual allele-A of rs6013897 in *CYP24A1* increased FPG levels by an average of 0.054 mmol/L. Moreover, the joint effects of rs739837 and rs6013897 on FPG indicated that carriers with more risk alleles showed much higher levels of FPG, with FPG increments of 0.082 mmol/L under the influence of risk allele (*P* = 0.003).

**TABLE 3:**
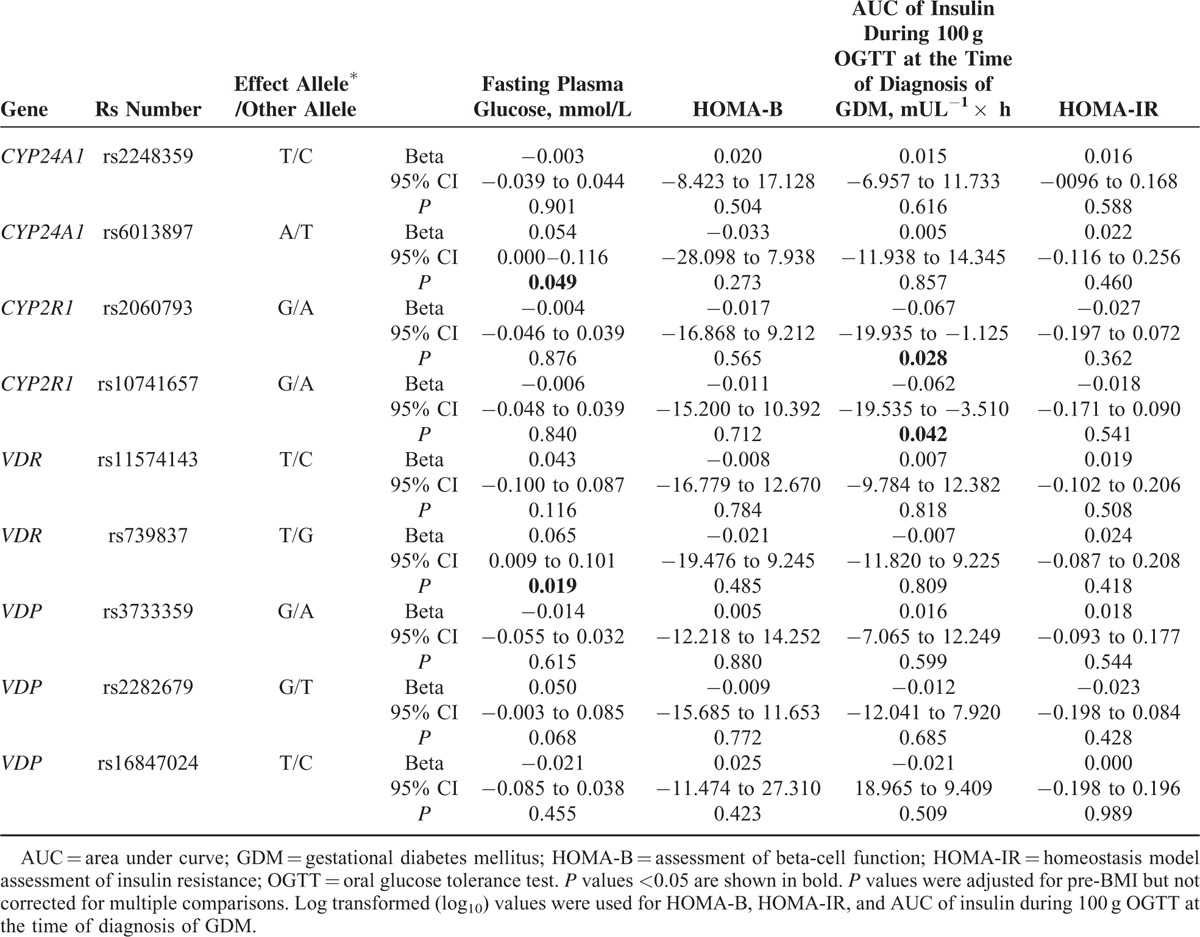
Associations Between Risk Alleles and Fasting Plasma Glucose, Insulin Beta Cell Function and Insulin Resistance

The risk alleles of rs2060793 and rs10741657 in *CYP2R1* were associated with reduced insulin AUC (B = −0.067 mU L^−1^ × h, *P* = 0.028, and B = −0.062 mU L^−1^ × h, *P* = 0.042, respectively), but not with HOMA-B. The combined effects of these loci also indicated that subjects carrying more risk alleles had a much lower AUC of insulin, with a 0.067 unit decrease per risk allele (*P* = 0.030). We did not detect any risk candidate locus for HOMA-IR.

We also observed an association between the loci and inflammatory factors (hs-CRP, white blood cell, and platelet count), and found that rs2248359 was associated with increased blood cell count (B = 0.063, *P* = 0.033), while rs16847024 was related to hs-CRP (B = 0.086, *P* = 0.005, Table 4).

**TABLE 4:**
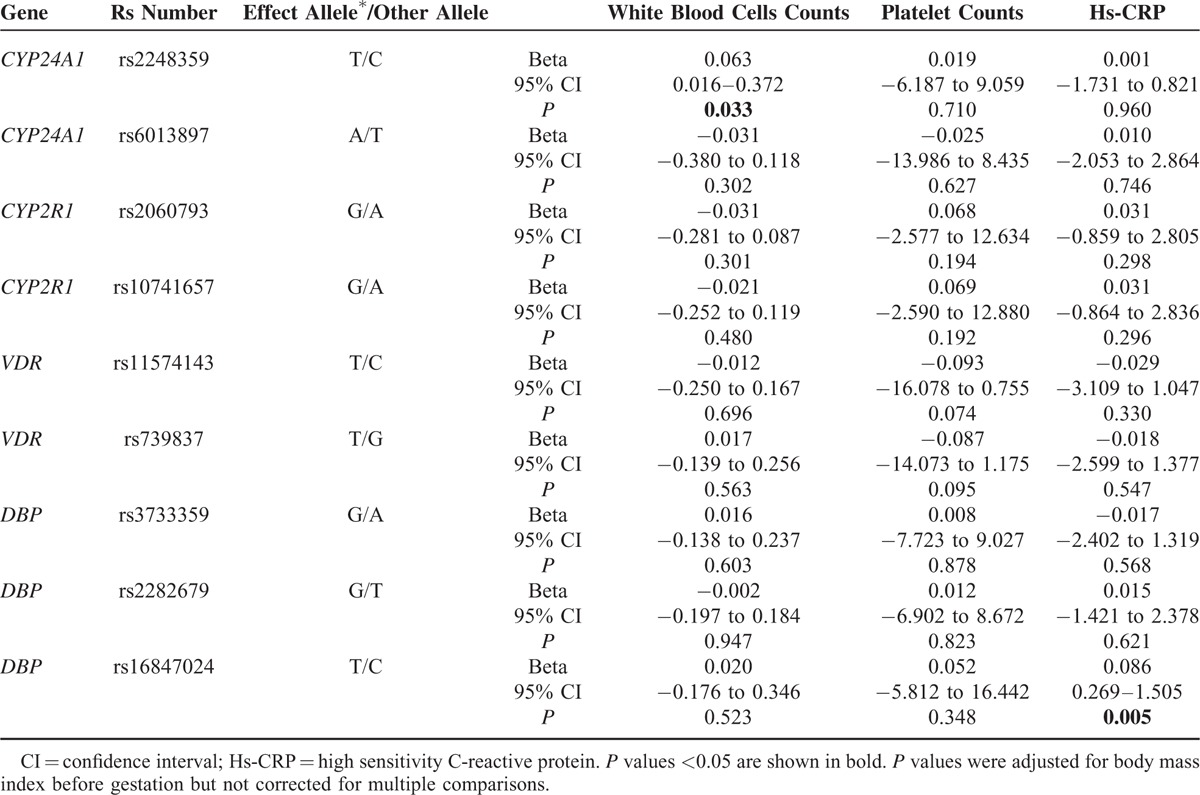
Associations Between Risk Alleles and Inflammatory Markers

## DISCUSSION

GDM is a disorder which caused by both genetic and environmental factors.^23,24^ It can be regarded as the early pathogenesis of T2DM and shares some physiological and genetic abnormalities that characterize T2DM. Genetic variants related to T2DM were found to be related with GDM.^25^

Vitamin D deficiency has been related with numerous health outcomes, including cancer, autoimmune disease, infectious disease, hypertension, heart disease, type 1 diabetes, type 2 diabetes, and GDM.^26^ The variations of gene involving in vitamin D production and metabolism are associated with concentration of vitamin D. In this study, we explored for the first time the relationship between variants involved in the production, degradation, and ligand-dependent signaling of vitamin D and GDM in a Chinese population. In addition, the association between quantitative traits connected with GDM and 9 representative loci was also analyzed. Polymorphisms in *GC* were found in association with GDM and several variants in *GC*, *CYP2R1*, *CYP24A1*, and *VDR* played roles in fasting glucose level, cell function, and inflammation.

### GC

Variants in *GC* have previously been reported in association with T2DM in Japanese and Polynesian Island populations.^10,27^ In the obese subgroup in the present study, the allele-G conferred protection against GDM at the rs3733359 locus. The haplotype analysis of *GC* indicated that haplotype-GG was lower in women with GDM compared with controls. Since the single allele (G) and haplotype (GG) were consistent in their protective effects, we concluded that the gene variations were at or near the functional level.^28^ Although variation in *GC* was not a major determinant of GDM, our data suggested that it may have a role in obese pregnant Chinese Han women.

*GC* variants were also related to quantitative traits connected with diabetes mellitus, including plasma glucose, insulin concentrations, and insulin resistance,^29,30^ an association which we did not observe in our study. However, we found a significant difference in hs-CRP among the groups based on rs16847024 genotypes, which may be in agreement with the previous finding that *GC* variants affected the immune response in different manners and resulted in distinct inflammatory conditions.^30^

### VDR

Many studies have reported the effect of *VDR* gene variants on T2DM,^10,31^ T1DM,^32^ obesity, and insulin secretion in response to glucose and FPG^33^; however, only a few studies have observed robust roles. In this study, we did not find an association between 2 loci (rs11574143 and rs739837) in *VDR* and GDM, but we observed that the variant rs739837 was related to FPG, consistent with previous research.^33^ Our results indicate that *VDR* was not a major candidate gene for GDM and insulin secretion. Of note, VDR itself is a transcription factor and regulates the transcription of other downstream genes in many tissues, including genes that are crucial for glucose metabolism. Although the rs739837 variant is not likely to influence the function of VDR itself since it is located in the 3′-untranslated region and does not impact amino acid sequence, it may be reside within a binding site of a microRNA capable of regulating VDR expression.

### CYP2R1

To date, *CYP2R1* has been investigated in patients with vitamin D deficiency and T1DM.^34^ In this study, we analyzed the role of 2 polymorphisms (rs2060793 and rs10741657) within the *CYP2R1* gene on susceptibility to GDM. Consistent with previous studies,^18^ we did not observe a correlation between rs2060793 and rs10741657 and GDM susceptibility; however, we found individual and combined effects of the variants on β-cell function, as estimated by AUC of insulin. The location of rs10741657 in 2-kb upstream of *CYP2R1* gene suggests that this polymorphism may affect the binding of transcription factors and then alter the level of vitamin D 25-hydroxylase expression, thus influencing the concentrations of 1, 25(OH)_2_D_3_ derived from 25(OH)D_3_,^34^ while 1,25(OH)_2_D_3_ had an effect on pancreatic β-cells by regulating CD8^+^ lymphocytes, macrophages, and interleukin-12.^35–37^ Therefore, our results provide evidence for the hypothesis that polymorphisms within *CYP2R1* could be functionally related to insulin secretion.

### CYP24A1

*CYP24A1* is responsible for the multiple side chain hydroxylation and/or oxidation in pathways leading to vitamin D inactivation in vivo.^5^ Numerous studies have investigated an association between *CYP24A1* and autoimmune disease-T1DM, but with conflicting results.^38,39^ It has been shown that subclinical inflammation is also an important risk factor for GDM.^20^ In the present study, we found that the risk allele of rs2248359 was associated with increased leukocyte count, although it showed no association with GDM. This phenomenon did not exist in another *CYP24A1* variant, rs6013897. That these 2 loci did not exhibit striking LD might be 1 explanation (D′ = 0.039, r^2^ = 0.000). In addition to its influence on inflammation, we also observed that FPG levels increased with increasing numbers of risk allele-A rs6013897. Based on these results, we speculate that the *CYP24A1* polymorphisms may play a role in inflammatory reactions and on the dynamic balance of blood glucose in GDM. To confirm this, further experiments investigating molecular and cellular actions of vitamin D and mechanisms of its protective effects in GDM are required.

The limitations of the present study need to be acknowledged. Firstly, the level of 25(OH)D was not measured in all subjects; therefore, we could not further evaluate the relationship between gene polymorphisms and GDM in different subgroups based on vitamin D levels. In fact, we observed decreased serum 25OHD concentrations in GDM patients compared with those in NGT pregnant women.^40^ Secondly, variants in *CYP27B1* were not interrogated, although *CYP27B1* is regarded as an important gene in the vitamin D metabolism pathway. Finally, the statistical power of the sample was of insufficient size to detect a small effect size (OR < 1.3); therefore, some weak associations may have not been detected.

In conclusion, the *GC* rs3733359 variant is associated with an increased risk of GDM in obese pregnant women. A subset of loci in *CYP2R1*, *CYP24A1*, *GC*, and *VDR* is related with β-cell secretion, fasting glucose, or subclinical inflammation. Such evidence is valuable in view of the limited research available on the genetic effects of GDM and could aid in identifying biomarkers for early risk prediction of GDM as well as the pathways involved in disease progression.
